# Supplementary material for: Analysis of PPARγ Signaling Activity in Psoriasis
Source: Int J Mol Sci. 2021 Aug 10;22(16):8603. doi: 10.3390/ijms22168603 (PMC8395241; doi:10.3390/ijms22168603)
Supplement: Supplementary file 1 [file ijms-22-08603-s001.zip › Supplemental materials_Analysis of PPARg signaling activity in psoriasis/Pathway models/Models images and html files/Model of PPARG related pathways in psoriasis (short version)/1012566762.html]

IRAK1 --+> TRAF6


# DirectRegulation IRAK1 --+> TRAF6

|  |  |
| --- | --- |
| URN | urn:agi-directregulation:in-out:urn:agi-llid:3654:out:urn:agi-llid:7189:eff=positive:mch=direct%20interaction |
| Connectivity | 2 |
| References | 909 |
| Effect | positive |
| Mechanism | direct interaction |
| ObjectType | DirectRegulation |
| RelationArity | 2 |
| RelationNumberOfReferences | 498 |
| RelationSymbolicName | IRAK1}OID --+> TRAF6}OID |
| RelationConfidenceLevel | 3 |
| RelationParticipantTypes | Protein -> Protein |
| Original # of References | 175 |

---
